# Dimer Is Not Double: The Unexpected Behavior of Two-Floor Peptide Nanosponge

**DOI:** 10.3390/molecules30010047

**Published:** 2024-12-26

**Authors:** Grazia Maria Lucia Messina, Marta De Zotti, Alvaro S. Siano, Claudia Mazzuca, Giovanni Marletta, Antonio Palleschi

**Affiliations:** 1 Laboratory for Molecular Surfaces and Nanotechnology (LAMSUN), Department of Chemical Sciences, University of Catania and Center for Colloid and Surface Science (CSGI), Viale A. Doria 6, 95125 Catania, Italy; gml.messina@unict.it; 2 Department of Chemical Sciences, University of Padua, Via Marzolo 1, 35131 Padua, Italy; marta.dezotti@unipd.it; 3 Laboratorio de Peptidos Bioactivos (LPB), Departamento de Química Organica, Facultad de Bioquímica y Ciencias Biologicas (FBCB), Universidad Nacional del Litoral (UNL), Santa Fe 3000, Argentina; asiano@fbcb.unl.edu.ar; 4 Department of Chemical Science and Technologies, University of Rome “Tor Vergata”, Via della Ricerca Scientifica, 00133 Rome, Italy; 5 Laboratory for Molecular Surfaces and Nanotechnology, (LAMSUN), Center for Colloid and Surface Science (CSGI), Viale A. Doria 6, 95125 Catania, Italy; gmarletta@unict.it

**Keywords:** peptide assembly, pH external stimuli, QCM-D, molecular dynamic simulation, functionalized surface, molecular sponge

## Abstract

Using the framework of an investigation of the stimuli-responsive behavior of peptide assembly on a solid surface, this study on the behavior of a chemisorbed peptide on a gold surface was performed. The studied peptide is a dimeric form of the antimicrobial peptide Trichogin GAIV, which was also modified by substituting the glycine with lysine residues, while the N-terminus octanoyl group was replaced by a lipoic one that was able to bind to the gold surface. In this way, a chemically linked peptide assembly that is pH-responsive was obtained because of the protonation/deprotonation of the sidechains of the Lys residues. Information about the effect of protonation/deprotonation equilibria switching the pH from acid (pH = 3) to basic (pH = 11) conditions was obtained macroscopically by performing Quartz crystal microbalance with dissipation monitoring (QCM-D), Surface Plasmon Resonance (SPR), Nanoplasmonic Sensing (NPS), and FTIR techniques. Using molecular dynamics (MD) simulations, it is possible to explain, at the molecular level, our main experimental results: (1) pH changes induce a squeezing behavior in the system, consisting in thickness and mass variations in the peptide layer, which are mainly due to the pH-driven hydrophilic/hydrophobic character of the lysine residues, and (2) the observed hysteresis is due to small conformational rearrangements from helix to beta sheets occurring mainly on the first half of the peptide, closer to the surface, while the second half remains almost unaffected. The latter result, together with the evidence that the layer thickness is not simply double the assembly of the monomeric analog, indicates that the dimeric peptide does not behave as a double monomer, but assumes very peculiar features.

## 1. Introduction

In recent years, peptide–based surfaces have gained considerable interest due to their potential applications in widespread fields, as they can be employed as drug delivery systems [1,2,3,4,5,6,7], photoelectric conversion devices [8,9,10], biosensors [3,11,12], nanostructured antimicrobial surfaces [13], and biomedical devices [1,14,15,16]. Peptides are indeed versatile molecules, easily chemically modifiable, whose structural and physicochemical properties can be tuned by an ad hoc arrangement of their sequence. In this contest, peptide systems are very relevant materials in the building of stimuli-responsive tools, as they can be synthesized to carry functionalities that make them sensitive to stimuli; the use of, for example, acidic or basic amino acids or bulky residues, means that the hydrophilic/hydrophobic balance in the peptide strands can be exploited to opportunely modulate the responsiveness of peptide-functionalized surfaces on stimuli that rule conformational changes and aggregation properties [1,3,10,14,17]. In this contest, for example, the responsiveness to a pH stimulus is exploited for the delivery of drugs in tumor cells more acidic than health cells or in several portions of the colon, which is characterized by different pH values, theragnostics of tumor cells, and the delivery of an electronic flux between redox centers [1,2,3,4,5,6].

In this framework, it is of fundamental importance to determine the key factors leading to the macroscopic features of the assembly to responsivity and to evaluate the stability of peptide-functionalized surfaces over use.

To this end, a characterization of the formation and stability over response cycles of peptide assembly on solid surfaces, a dimer of an analog of the Trichogin GA IV (GA IV), was performed. GA IV is a very peculiar natural peptide with antimicrobial functions. It has the sequence Oct-Aib-Gly-Leu-Aib-Gly-Gly-Leu-Aib-Gly-Ile-Lol, where Oct and Lol are the octanoyl and leucinol moieties, respectively, and Aib is α-amino-isobutyric acid, a non-usual C^α^ tetrasubstituted amino acid.

**Scheme 1:**
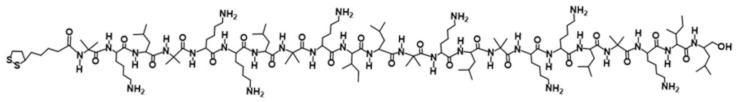
The chemical structure of the peptide L2.

GA IV has been thoroughly characterized by the authors in both solutions and interfaces using physicochemical techniques and molecular dynamic (MD) simulations [18,19], showing that even though it is relatively short (11 amino acids long), it adopts a helical structure in both its solution and membrane phase, forming aggregates characterized by small conformational rearrangements. This is because the presence of this tetrasubstituted C^α^-atom in the Aib residue restricts the available conformational space, also stabilizing the helical structures in very short oligopeptides. Furthermore, the presence of this unusual residue makes the peptide less prone to enzymatic degradation. In detail, the analog investigated here has the formula Lipo-Aib-Lys-Leu-Aib-Lys-Lys-Leu-Aib-Lys-Ile-Leu-Aib-Lys-Leu-Aib-Lys-Lys-Leu-Aib-Lys-Ile-Lol (L2, Scheme 1). It is a dimeric analog of GA IV in which each glycine amino acid has been substituted with lysine residues and the octanoyl group has been replaced by a lipoic group. The presence of lysine residues ensures the pH-responsiveness of the peptide systems because Lys is a basic amino acid (pKa = 10.73 [20]) while the lipoic group, which contains two sulfur atoms capable of binding to the gold surface, ensures the linkage of the peptide to the surface (Scheme 1). Furthermore, the replacement of Gly, the most flexible amino acid, with Lys, a bulkier one, in L2 favors folded conformations (at high pH).

In a recent article [21], we described the factors driving the sponge-like squeezing behavior—that is, the stretching and shrinking behavior of the chemisorbed layers of Lipo-Aib-Gly-Leu-Aib-Gly-Gly-Leu-Aib-Gly-Ile-Lol, a monomeric GA IV analog (having the same kind of substitution of L2) on the Au surface as a function of pH changes. In this case, we have demonstrated that a combination of experimental and theoretical studies cannot be explained simply by considering a stretching–contraction intrachain electrostatic effect due to the pH-driven charging and uncharging of theNH_2_ group of the Lys sidechains.

In fact, we have shown that the cyclic simultaneous change in the important features of the studied system (mass, thickness, and homogeneity) is more accurately interpretable because it is due to an extended interchain aggregation processes caused by the pH-induced switching of the hydrophilic and repulsive character of charged Lys groups to a more hydrophobic one of the same uncharged groups along the peptide chain. The results demonstrate that the manifoldness of the macroscopic features due to the pH change could be reduced to a few critical factors leading to intrachain structural processes, in turn promoting dynamical assembly/disassembly interchain processes. This means that a simple pH-driven conformational change mechanism is not the leading one. Furthermore, the squeezing behavior of this assembly has been found to be fully reversible in pH cycling.

Based on this result, it is expected that the assembly of L2 on gold gives a similar behavior when changing the pH.

In this case, in fact, the main characteristics (amphipaticity, presence of Aib residues, pH responses, etc.) of the monomeric analog are maintained, doubling only the sequence. The results presented here indicate that this is not exactly the case, and the studied assembly is not simply a double-thick monomer assembly. Interestingly indeed, QCM-D experiments indicate that even if the L2 assembly shows a pH-dependent squeeze behavior, as the monomer does, a hysteresis process occurs when subsequent pH changes are performed. MD simulations of the L2 assembly on gold, including four pH change cycles (each composed of a monolayer simulation at pH = 3, followed by one simulation at pH = 11), have been performed to explain, at the molecular level, this effect, allowing u to highlight the role of the increased conformational degrees of freedom expected for such longer, yet very similar, biomolecules with respect to the monomeric GA IV analog. In other words, this study focuses on the evaluation of the stability of the dimeric peptide assembly over repeated cycles of pH changes, which is a key feature for its potential applications, and on the molecular features leading to an irreversible macroscopic rearrangement of the assembly. The results indicate that L2 on gold behaves like a “two floor” peptide, in which each of the halves assumes a different rearrangement. The first, closer to the surface, indeed, tends, during pH cycling, to change conformations and to form aggregates irreversibly, while the second half, further from the surface, remains almost unaffected.

## 2. Results and Discussion

### 2.1. Quartz Crystal Microbalance with Dissipation Monitoring and Nano Plasmonic Sensing Measurements

#### Assembly on Surfaces

The assembly of peptide L2 on the gold surface was monitored by means of Quartz crystal microbalance with dissipation monitoring (QCM-D) experiments.

The very low dissipation value obtained, together with the absence of significant overtone spread, indicates that the L2 forms a rigid layer, strongly bound to the gold surface, as demonstrated by repeated rinsing steps [21]. These results allow us to apply the Sauerbrey equation to calculate the uptake of the hydrated peptide mass on the gold surface (the so called “wet mass”) in the steady state (see Section 3.2) [16].

At the same time, surface plasmon resonance (SPR) measurements are useful for obtaining, from the experimentally observed plasmon shifts, the thickness of the peptide layers in air. It should be noted that the SPR technique measures the optical mass of the adsorbed material, so the estimated thickness is related to the so-called “dry mass”. Table 1 reports the results obtained using QCM-D and SPR regarding the characterization of the adsorbed layer on the gold sensor of the L2 peptide.

### 2.2. Nanosponge System

The Nano Plasmonic Sensing (NPS) technique allows us to obtain an estimate of the peptide layer thickness variations by changing the pH solution.

**Figure 1:**
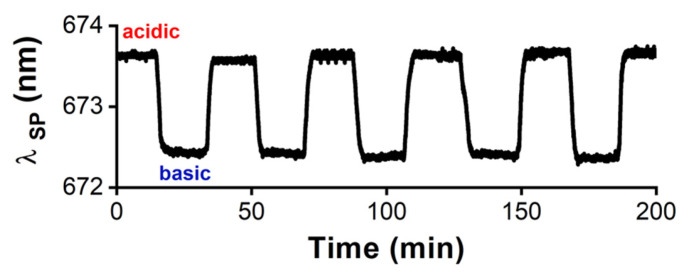
NPS data showing the change in resonance plasmon, that is, the thickness change, for the L2 peptide layer with the change from pH = 3 (starting baseline) to pH = 11.

Figure 1 reports the NPS results, showing regular cycling. It should be noted that when a basic solution was injected into the L2 peptide layer, there was a blue-shift variation in surface plasmon resonance (∆λ) of approximately 1.24 nm. On the other hand, when the acid solution was switched on, a red-shift in the same magnitude was found. This process is completely reversible as observed upon following multiple pH changes cycles, and this suggests that this process is attributable to the electrostatic effects arising from the pH-induced protonation/deprotonation of the Lys sidechains. The change in thickness from acid to basic pH is 1.32 ± 0.06 nm, obtained by applying Equation 1 (see Section 3.3).

QCM-D analysis has been performed to study the effect of pH on the mass uptake.

**Figure 2:**
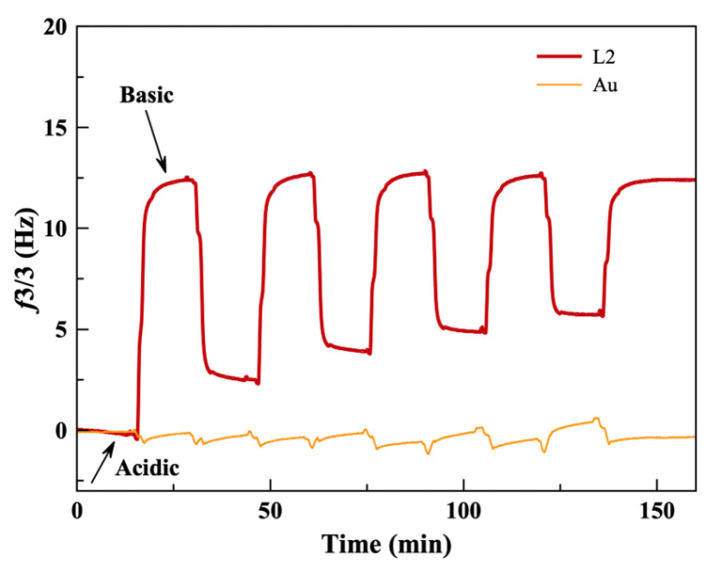
QCM-D data showing the cyclic behavior of frequency increase (basic condition) and recovery (acid condition) of the L2 monolayer (red) and of gold (yellow) by changing the pH. The baseline is set for the sample in an acid environment at pH = 3.

Figure 2 shows that the QCM-D response on the bare Au crystal remains constant, whereas the L2 layer exhibits a remarkable (although partial) cyclic reversible resonance frequency shift by changing the pH from acid to basic. In detail, we found an increase in the frequency shift (index of a mass loss) under a higher pH condition and, after the pH was lowered, a recovery of the starting frequency resonance. This behavior corresponds to a cyclic loss and uptake of mass in the peptide layer adsorbed to the surface, which, according to MD simulations (see Section 2.3), involves water molecules and ions [21,22].

**Figure 3:**
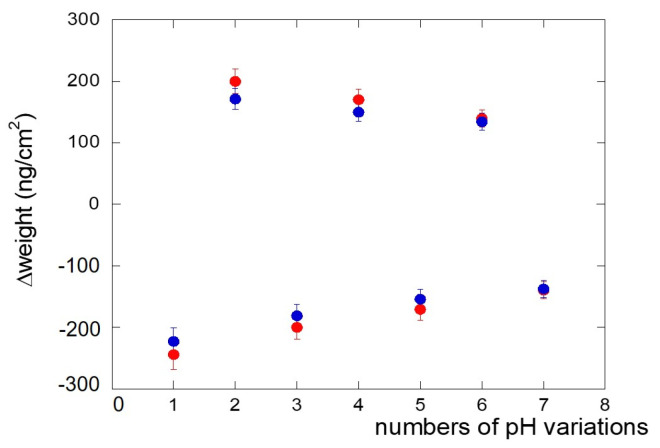
Weight loss/gain after each pH variation obtained using the QCM-D experimental data (blue circles) and MD simulations (red circles). See Section 3 for details.

However, it seems important to stress that this process, studied with this technique, is not fully reversible, but shows unexpected hysteresis, as reported in Figure 2 and highlighted in Figure 3, in which the mass loss in the system for each acidic to basic pH change and the related mass gain followed for each basic to acidic pH change is reported. Moreover, note that this hysteresis does not influence the NPS measurements that detect the layer thickness. Therefore, this effect cannot be related only to the amount of water mass uptake. The data from MD simulation explain the nanosponge mechanism, which is connected to the different structural rearrangements of the peptides.

### 2.3. MD Simulations

The experimental results reported above essentially point out several important macroscopic features induced by the pH switching, namely, the thickness variation and the counter-correlation of the resonant frequency of the L2 assembly (index of a mass loss/gain cycle). We suggested above that the unusual counter-correlation of the two phenomena may stem from a complex change in the features of the peptide assembly induced by the pH cycling, which in turn provokes measurable changes in their mechanical properties. Furthermore, another (new) peculiar aspect that needs to be understood is the slight hysteresis of the resonant frequency obtained at acid pH during QCM-D experiments. In detail, hysteresis implies, macroscopically, a growing loss of mass every time the pH decreases. To evaluate this point and furnish details at the molecular level of the system under investigation, such as, for example, mass changes and peptide aggregation features, MD simulations were performed. The MD results allow us to explain changes in the system due to pH cycling at the nanoscopic scale that determine the overall macroscopic changes in the monolayer features at longer time scales. To this end, eight molecular dynamic simulations of the system were performed, simulating four pH change cycles (each composed of a monolayer simulation at pH = 3, followed by one simulation at pH = 11).

#### Effects on the Monolayer Due to the Variation in pH

**Figure 4:**
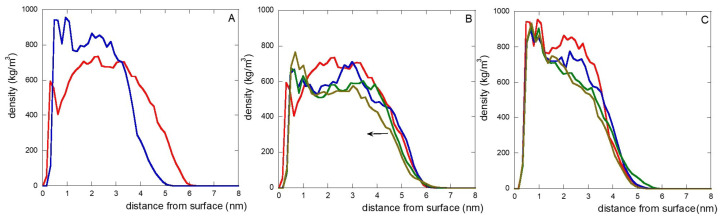
Peptide density as a function of distance from the surface of (**A**) for the first cycle (pH = 3 (red) and pH = 11 (blue)), (**B**,**C**) for the subsequent cycles (I: red; II: green; III: blue; IV: brown), (**B**) at pH = 3 and (**C**) at pH = 11. Arrows indicate the trend of peptide thickness.

First, MD calculations on 64 L2 molecules, placed on a gold surface in a simulation box (MD experimental details in terms of, for example, simulation parameters, time scales, vdW cut-off, etc., are described extensively in Section 3.5—Molecular Dynamics Simulations, reported below), were performed to evaluate the peptide density for L2 monolayer at pH = 3 and pH = 11 on the gold surface after equilibration state. As shown in Figure 4A, peptide density was found to be much different with the change in pH.

**Figure 5:**
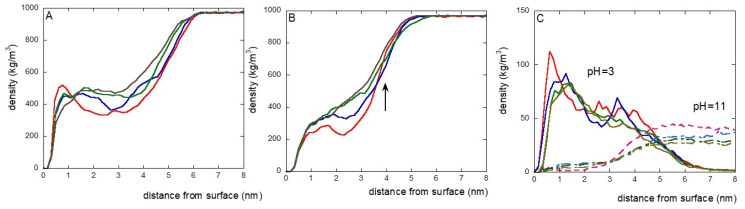
Water density as a function of distance from the surface for the simulations at pH = 3 (**A**) and at pH = 11 (**B**); (**C**) ion density as a function of distance from the surface for the simulations at pH = 3 (continuous lines) or pH = 11 (dashed lines). Colors of the cycles: I: red; II: green; III: blue; IV: brown. Arrow is a guide for the eyes.

**Figure 6:**
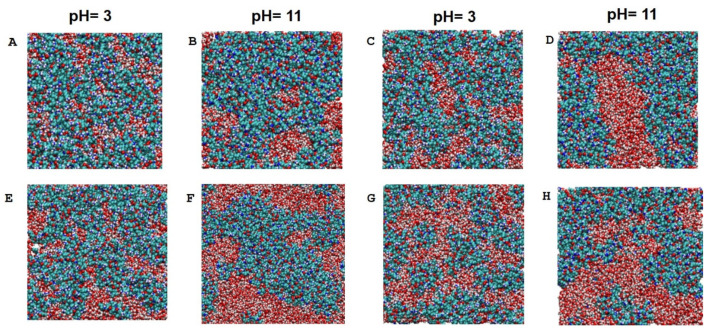
Top view snapshots of the last frame of the eight MD simulations concerning the four pH change cycles: (**A**,**B**) I cycle; (**C**,**D**) II cycle; (**E**,**F**) III cycle; (**G**,**H**) IV cycle). (**A**,**C**,**D**,**F**) are related to the monolayer at pH = 3; the others are related to the monolayer at pH = 11. Oxygen atoms are in red, hydrogen atoms are in white, nitrogen atoms are in blue, and carbon atoms are in cyan.

At lower pH, the density of the peptides tends to zero at about 6.4 nm from the gold surface, while under basic conditions, it occurs at a lower distance, approximately 5.6 nm, in good agreement with the NPS data reported in Table 1, thus confirming the formation of the assembly (also, the initial weight obtained, considering the peptides and connected solvent molecules, is approximately 900 ng/cm^2^, very close to the experimental value reported in Table 1). Furthermore, the maximum value obtained for the peptide density is much higher at pH = 11 (approximately, 103 kg/m^3^ at about 1 nm from the surface) than at acidic pH (approximately, 700 kg/m^3^ at about 3 nm from the surface). At the same time, also, the water density has different profiles at the two investigated pH values, as shown in Figure 5A,B. Ions are also present up to 6 nm from the surface at pH = 3, thus being close to the peptide moiety, while at pH = 11, they are located mainly in bulk water, away from the peptide layer (ion density value begins to increase at distances greater than 3 nm from the gold surface) (Figure 6C).

It is interesting to note that the overall profile also looks different, indicating a different structural rearrangement of the peptides on the surface when varying the pH of the system, as also shown in Figure 6.

Snapshots of simulations of the in-plane distribution of the peptide and water molecules at the two different pHs (Figure 6) indicate that the uniformity of the peptide layer is strongly affected by pH, while at pH = 3, water molecules surround the peptides almost uniformly; at pH = 11, they are confined mainly in “pools”, and the peptides tend to aggregate among themselves.

Overall, these data indicate that a drastic peptide shrinkage, and a consequent water (and ions) depletion from the layer, occur on increasing of the pH. The good agreement between the experimental (QCM-D data) and MD results regarding water and ion mass gain and loss due to the pH changes, reported in Figure 3, supports these conclusions. Furthermore, MD experiments confirmed and provided a molecular explanation for the unexpected result found using QCM-D experiments, that is, that the pH cycling yields an almost reversible feature with a hysteresis for the mass recovery of the L2 monolayer every time the pH decreases. As the number of pH cycles increases, indeed, the characteristics of the peptide density profiles at pH = 3 (Figure 4B,C), and, consequently, those of the water density (Figure 5B,C), change slightly toward a formation of smaller peptide agglomerates surrounded by water (Figure 6).

**Figure 7:**
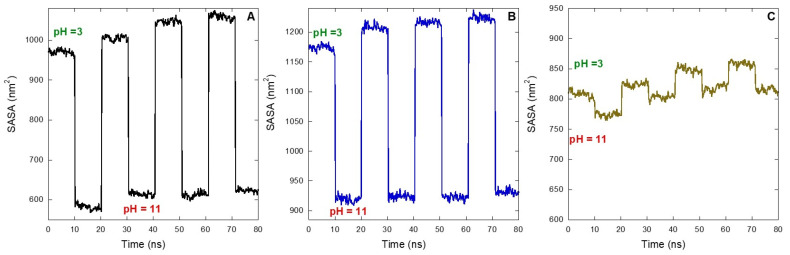
SASA values of the last 10 ns of MD simulations, during several cycles of pH variation (pH = 3 → pH = 11). (**A**) The peptide moieties; (**B**) the LYS side chains; (**C**) hydrophobic (Leu, Ile, and Aib) side chains.

This feature is confirmed by Solvent Accessible Surface Area (SASA) analysis performed on peptides upon changing the pH. As shown in Figure 7A, in fact, the SASA of the peptide moiety is double at pH = 3 than at pH = 11. This is due to the charging and discharging of Lys residues as a function of pH, as clearly shown in Figure 7B, in which the SASA values of the Lys side chains alone are reported. Indeed, in acidic conditions, the charged groups of Lys protrude towards water, and thus peptides are surrounded by water. In contrast, at pH = 11, the absence of charges makes the interchain aggregation more favorable and the peptides tend to aggregate excluding water from their assembly. Consequently, water molecules tend to confine themselves, as explained earlier, in relatively large pools in the system. In fact, the hydrophobic moiety (Aib, Ile, and Leu side chains) of the peptide are not involved in the pH-dependent squeezing mechanism, as demonstrated by the very low SASA changes as a function of pH (Figure 7C). On performing MD simulations of the system subjected to subsequent pH change cycles, it is found that an increase in the SASA values of the Lys side chains (and, accordingly, of the peptides) at pH = 3 (Figure 7A,B) occurs.

**Figure 8:**
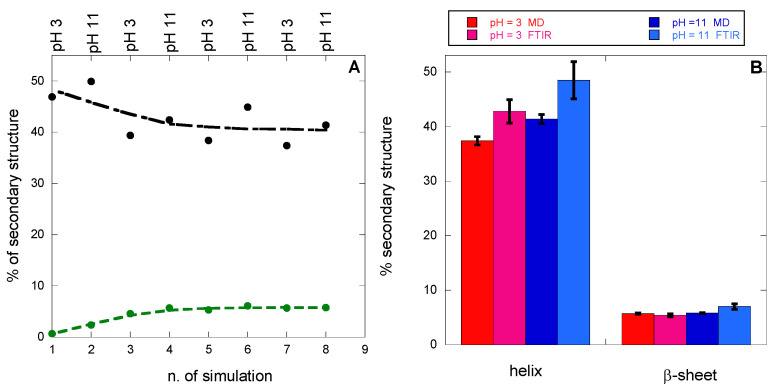
(**A**) Percentage of secondary structures (black: helix, and green: β-sheets) upon the eight simulations mimicking the four pH cycles (pH = 3 → pH= 11). (**B**) Comparison of the percentages obtained using MD simulations and FTIR experiments of secondary structures at pH = 3 (red and violet, respectively) and at pH = 11 (blue and light blue, respectively).

It is interesting to note that, on subsequent pH cycles, small but significant rearrangements of peptides occur, as demonstrated by a peptide conformation analysis from MD. This means that the peptide conformation changes slightly in cycling, and hysteresis is associated with an irreversible initial decrease in the helical content and an increase in β-sheet structures, suggesting an irreversible different packing of peptides (Figure 8A). The formation of β-sheets structures has been a surprising finding, because it is well known [18,19,23,24] that peptides carrying Aib tend to assume helical conformations, but this result has been confirmed by FTIR experiments, performed in reflection absorption spectroscopy (RAS) mode, whose results are reported in Figure 8B, which indicate that, at the last pH cycles, a small amount (less than 10%) of β-sheets occurs (localized at 1615–1635 cm^−1^ in the FTIR spectra, while helices are in the 1650–1670 cm^−1^ region) [25,26] and its percentage is slightly higher at pH = 11.

Overall, the experimental and theoretical results indicate that the squeezing mechanism, being the changes in layer features (thickness and mass) due to pH switching, is mainly caused by the pH-driven hydrophilic/hydrophobic character of the Lys residues. At lower pHs, indeed, they are charged and therefore assume a hydrophilic character, while under basic conditions, they are uncharged and assume a prevalent hydrophobic character. This variation leads, in turn, to modifications of the layer behavior in terms of peptide aggregation, which favors the formation of a more homogeneous layer at pH = 3, in which the peptides are surrounded by water due to the outward exposure of the groups to water. At pH = 11, instead, changing the character of the lysine groups tends to aggregate, resulting in a local layer shrinking and depletion of trapped water, which is then segregated in pools of water.

In addition, to obtain a molecular characterization of the observed hysteresis process, we performed a conformational analysis by dividing the L2 peptide into two halves, each corresponding to the GA IV.

**Figure 9:**
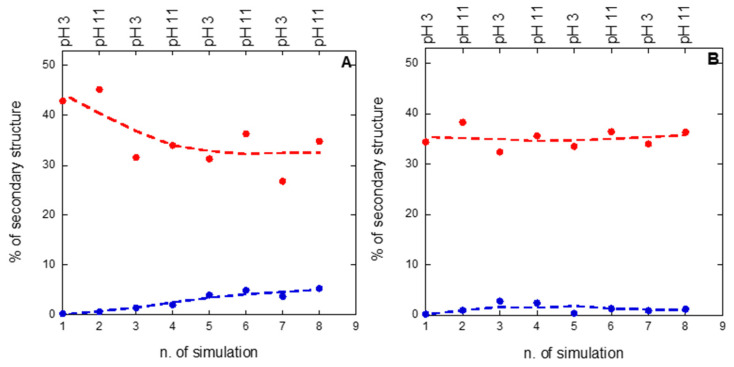
Percentage of secondary structures (red: helix; blue: β-sheets) upon the eight MD simulations corresponding to the four pH cycles (pH = 3 → pH = 11) for (**A**) the first half (closer to the surface) and (**B**) the second half (further from the surface) of the L2 peptide.

As shown in Figure 9, with increasing pH cycles, the first half of L2, closer to the surface, undergoes a conformational change that decreases the percentage of helical structures by approximately 20%, which are transformed into β-sheets, up to about 4.5%, and coils (Figure 9A). On the contrary, the second half of L2, further from the surface and closer to the aqueous phase, does not show substantial conformational changes with increasing pH cycles (Figure 9B).

This result allows for the rationalization of the experimentally observed hysteresis, essentially due to the tract near the surface, which, during the cycles, modifies the starting conformation resulting from the first time the monolayer meets the acidic solution. On the contrary, the second half of L2, further away from the surface, substantially maintains the conformation of the first experiment. Note that, after the initial cycles, the local conformational variations tend to repeat themselves and, as already reported, this process depends only on the approaching or distancing of the Lys side chains at acidic or basic pHs, respectively.

**Figure 10:**
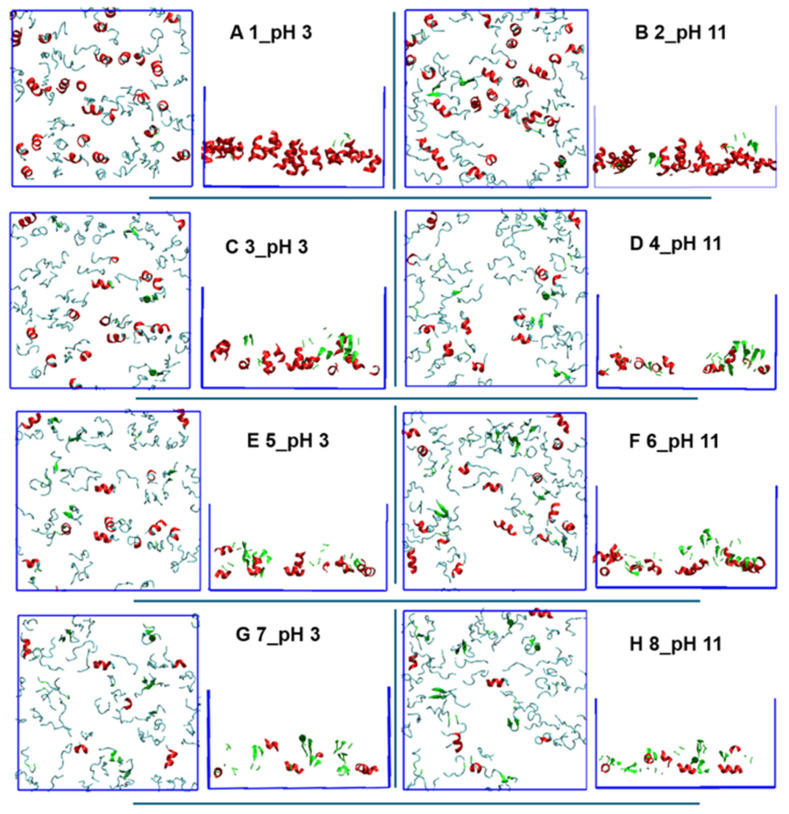
In each panel, a top view (left) and side view (right) of the last frame of the eight MD simulations (**A**–**H**) corresponding to the four pH cycles (pH = 3 → pH = 11) of the L2 half portion closer to the surface are presented. In the snapshots, only β-sheets and helix structures (side view) and coils (top view only) in peptides are shown. Helical tracts are in red, β-sheets are in green, and coils are in cyan.

**Figure 11:**
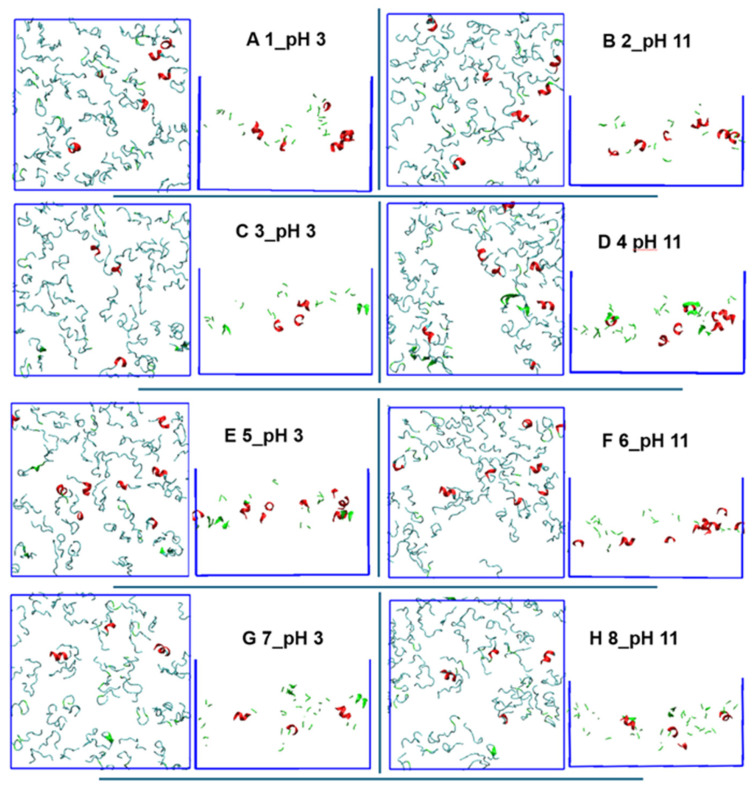
In each panel, a top view (left) and side view (right) of the last frame of the eight MD simulations (**A**–**H**) corresponding to the four pH cycles (pH = 3 → pH = 11) of the L2 half portion further from the surface are presented. In the snapshots, only β-sheet and helix structures (side view) and coils (top view only) in peptides are shown. Helical tracts are in red, β-sheets are in green, and coils are in cyan.

These observations indicate that, upon cycling, an overall layer rearrangement occurs to reach a stable structural state, mainly due to the first portion of the peptide, closer to the surface and optimize the contact between peptide chains and water molecules present in the inter-peptide spaces, which leads concomitantly to a decrease in the number of water molecules linked to peptides and an increase in the amount of bulk free water. It can be assumed that, during a cycle, local conformational variations (i.e., transition from helix to β-sheet) occur which, in turn, influence peptide–peptide interactions (in terms of the Lys–Lys and Lys–water interactions) of the whole monolayers, affecting the monolayer rearrangements in the successive pH cycles (as shown in Figure 6). In other words, at pH = 11, the hydrophobic effects predominate and the peptide aggregate. Upon decreasing the pH, the electrostatic interactions, due to the presence of charged Lys side chains, result in peptide disaggregation. On returning to pH = 11, the aggregation of peptides occurs again, but, plausibly, the system is affected by the local optimized rearrangements (formation of β-sheets) obtained in the previous step. Thus, every pH change can be considered to be a perturbation step towards the stabilization of the monolayer. A clarifying scheme of process is reported in Figure 10 and Figure 11, in which the occurrence of the first β-sheet structure, occurring on the first pH cycle, is retained (and increased) on the subsequent cycles.

## 3. Materials and Methods

### 3.1. Synthesis and Characterization of Peptide

L2 was synthesized by manual solid-phase peptide synthesis, exploiting the procedure already published [21] with a yield of 60%. The peptide purity was over 99%. ESI-MS: [M + 2H]^2+^ expected = 1316.9065; [M + 2H]^2+^ found = 1316.9388; [M + 3H]^3+^ found: 878.2893; [M + 4H]^4+^ found: 658.9594.

Fluorenyl-9-methyloxycarbonyl (Fmoc) amino acids and all other amino acid derivatives and reagents useful for peptide synthesis were purchased from Merck (Merck KGaA, Darmstadt, Germany). O-(7-Azabenzotriazol-1-yl]-1,1,3,3 tetramethyluronium (HATU) hexafluorophosphate and 7-aza, 1-hydroxy-benzotriazole (HOAt) were purchased from GLS (Shanghai, China). Assembly of peptides on resin was performed on a 0.06 mmol scale, exploiting single coupling (55 min long, dimethylformamide (DMF) as solvent) mediated by O-benzotriazol-1-yl]-1,1,3,3 tetramethyluronium (HBTU) hexafluorophosphate, 1-hydroxy-benzotriazole (HOBt), and N,N′diisopropylethylamine (DIPEA). The deprotection of Fmoc group was performed using a 20% piperidine solution in N,N-dimethylformamide. The coupling steps involving Aib residues, carried out in presence of HATU, were doubled. The lipoic moiety at the N-terminus was introduced using the preformed activated ester of lipoic acid (obtained by reaction with an equivalent amount of N-[3-(dimethylamino)-propyl]-N′-ethylcarbodiimide (EDC) and HOAt) in the presence of N-methylmorpholine. The coupling procedure took 1 h and was repeated twice. Cleavage of peptide from the chlorotrityl resin was obtained using 1,1,1,3,3,3-hexafluoroisopropanol (HFIP) 30% in dichloromethane. The filtrate was collected, and this step was repeated three times. The solution was then concentrated under a nitrogen flow. The crude peptides were purified using a medium-pressure purification system Isolera Prime (Biotage, Uppsala, Sweden) and characterized using an analytical RP-HPLC on a Vydac C18 column (4.6 × 250 mm, particle size: 5 μ, pore size: 300 Å) using a Dionex (Sunnyvale, CA, USA) P680 HPLC pump with an automated sample injector ASI-100 and spectrophotometric detection (flow rate 1.0 mL/min; λ = 226 nm; room temperature) with a binary elution system: A, 0.1% trifluoroacetic acid (TFA) in H_2_O; B, 0.1% TFA in CH_3_CN/H_2_O (9/1 *v*/*v*); gradient 50–90% B in 30 min.

Electrospray ionization (ESI-MS) was performed on a PerSeptive Biosystem Mariner instrument (Framingham, MA, USA).

### 3.2. Quartz Crystal Microbalance with Dissipation Monitoring (QCM-D)—Peptide Monolayer Formation

Peptide layer formation was performed by using gold-coated sensors in a Q-Sense E1 instrument (Biolin Scientific, Göthenburg, Sweden).

The gold-coated sensors were cleaned before use, performing 10 min of UV-ozone treatment (Jelight Company λ_exc_ = 185 and 254 nm), followed by rinsing with ethanol and drying under a stream of nitrogen.

QCM-D experiments were performed to follow both the formation of the peptide layer, and the effect of the pH change in the solution on the chemically linked peptide layer on gold. In particular, to study the peptide monolayer formation, a 1 mM ethanol peptide solution was injected in QCM-D apparatus under steady laminar flow conditions (150 μL/min), and the peptide binding process was followed for 18h until reaching the steady state. Rinsing steps with ethanol were performed on the steady-state peptide layer to remove all molecules not chemically or weakly bound on the gold sensor surfaces. To study the pH response of the peptide layer, an HCl 10^−3^ M solution at pH = 3 was at first used for the stabilization of the baseline. After stabilization, a NaOH 10^−3^ M solution at pH = 11 was injected and the behavior of the system was followed for 15 min, and then the solution was replaced with that at pH = 3, and the behavior of the system was then monitored again for 15 min. This pH cycle was repeated several times.

The QCM-D measurement were performed for the fundamental resonance frequency (*n* = 1, i.e., f = 5 MHz) and the six overtones (n = 3, 5, 7, 9, 11, and 13, corresponding to f = 15, 25, 35, 45, 55, and 65 MHz, respectively). In the cases of rigid, evenly distributed, and sufficiently thin adsorbed layers, the frequency to mass conversion was simply obtained by using Sauerbrey equation [22], ΔM = −(C/n)·f, where f is the decrease in resonant frequency, M is the mass uptake at the sensor surface, C is a constant depending on the intrinsic properties of quartz slab (in our case C = 17.7 ng/cm^2^ Hz^−1^ at f = 5 MHz), and *n* (*n* = 1, 3) is the overtone number. The resolutions in f and D are ±0.1 Hz and 1 × 10^−7^, respectively [27]. Since the ratio between dissipation and frequency is less than 4 × 10^−7^, the Sauerbrey equation was applied, consisting of the average relative to the frequency values before the peptide solution reaches the sensor and after the adsorption process (18 h). Knowing the used concentration of peptide and the peptide molecular weight, it is possible to calculate the number of molecules per cm^2^.

### 3.3. Surface Plasmon Resonance (SPR) and Nanoplasmonic Sensing (NPS) Technique

The peptide thickness on gold substrates was obtained by monitoring the optical plasmon variations at the surface of SPR gold coated sensor using a standard SPR Navi200 (BioNavis Ltd., Tampere, Finland) equipped with 670 nm light source. Experiments were performed at 25 °C in air, and all the samples were measured before peptide deposition (considered as a reference) and after the peptide layer deposition. The produced angular spectra were then analyzed using BioNavis Layer Solver software. The thickness of the peptide layer was obtained in air; to this end, the refractive index of peptides was determined by applying the approach of H. Zhao et al. [28], taking into account the physico-chemical properties of amino acids constituting the peptides. In this way, the calculated refractive index is 1.522, very close to the experimentally measured values for peptides [29].

Ensemble-averaged Nano Plasmonic Sensing (NPS) measurements were performed in optical transmission mode using an Insplorion XNano instrument, and sensor chips from Insplorion AB (Gothenburg, Sweden); they are gold nanodisks deposited onto glass slides with random arrangement (surface coverage ≈ 7%), prepared using hole-mask colloidal lithography. Each gold nanodisk is characterized, on average, by a height and diameter of about 25 and 150 nm, respectively. The bulk sensitivity of the nanodisks obtained by experiments using water/glycerol mixtures was approximately 150 nm per refractive index unit.

In order to study the effects of pH’s changes on the thickness of the peptide layers anchored to the gold nanodisks, the NPS response of the coated sensor was recorded in two different solutions at pH = 3 and pH = 11, respectively.

According to the model of finite and homogeneous thickness having the refractive index of the material at the gold interface, the variation in the NPS peak wavelength (∆λ) depends linearly on the difference in refractive index between the peptide adlayer and the solution (n_peptide_ and n_sol_) and exponentially on the thickness of peptide layer (t) using the following Equation (1) [30]:

Δλ = S [1 − e ^−t/L^] (n_peptide_ − n_sol_) (1)

where S = 150 nm/RIU is the bulk refractive sensitivity of the NPS sensor, L = 30 nm is the decay length of the NPS evanescent wave according to the sensor’s specification, t is the adsorbed peptide layer thickness, and n_peptide_ = 1.522, n_sol_ = 1.335, and Δλ are the difference between the centroid λ values before and after the pH changing solution.

The baseline for the NPS response was measured in the lower pH solution. After the stabilizing the signal, the pH = 3 solution was replaced by the basic one (pH = 11), and the behavior (in terms of Δλ max) of the peptide layer at the sensor surface was monitored as a function of time. To test the reversibility of our system, for each measurement, the pH cycle (that is, the switch from acid to basic solution) was repeated several times. During the experiments, the solution was continually introduced by means of a peristaltic pump (constant flow rate = 50 μL/min).

Data analysis was performed using the Insplorer software package (Insplorion AB, Gotenborg, Sweden). The time resolution was 1 Hz, and the centroid position and the related spectral resolution of the plasmon resonance were determined using high-order polynomial fitting [31].

### 3.4. Fourier Transform Infrared Reflection-Absorption Spectroscopy (FTIR-RAS)

FTIR-RAS spectra were obtained using a Thermo-Fisher (mod. Is50) instrument (Thermo Fisher Scientific Inc., Madison, WI, USA) equipped with a Veemax TM III Variable Angle Reflectance Accessory (Pike Technologies, Madison, WI, USA) and a liquid nitrogen cooled mercury–cadmium–telluride (MCT) detector. Experiments were performed collecting for each spectrum, 256 scans, with a resolution of 2 cm^−1^, using an incident beam at an incidence angle of 80° with respect to the sample surfaces. Samples were prepared the same as for QCM measurements. The dried samples were placed using a homemade gas-tight cover under D_2_O atmosphere for 120 min [21,24] to allow for the exchange of H_2_O with D_2_O. A value of 80% relative humidity (RH) in the chamber was reached, as measured with a Delta Ohm hygrometer (Delta Ohm Srl, Padua, Italy), mod. HD 8901). Identification of peptide secondary structures was obtained by performing a deconvolution of bands in the Amide I region (1750–15,900 cm^−1^) using the OMNIC software (version 9.12.958), considering it to be a sum of Gaussian functions, whose FWHH was constrained to be below 25 cm^−1^ [26].

### 3.5. Molecular Dynamic Simulations

Simulations were performed using the Gromos53a6 force field present in the Gromacs 2024.3 software [32,33,34]. The parameters for the Aib residue and the lipoic and leucinol moieties were already reported [18,21], while the gold non-bonded interactions were described according to Pu et al. [35]. The simple point charge SPC model was used for describing the water.

To maintain the temperature constant, a velocity-rescale thermostat, with a coupling constant of 0.6 ps, was applied, while a C-rescale isotropic barostat was employed with a time constant of 1 ps to keep the pressure constant. The constraint on the length of the bonds was obtained with the LINCS algorithm [36]. Electrostatic interactions were calculated using the particle mesh Ewald procedure (cutoff = 1.4 nm) [37,38], and van der Waals interactions were obtained using a cutoff radius of 1.4 nm. In regard to the surface, the initial configuration of the gold layer was a flat surface of Au(111) atoms.

All simulations were performed according to our usual protocol [21], where a 100 ns production run with a time step of 2 fs was preceded by an initial energy minimization, during which the peptide atoms were positionally constrained, and then by a 100 ps equilibration run with a time step of 0.5 fs.

The initial configuration of the first simulation at pH = 3 was obtained using the following procedure. In total, 64 L2 molecules with uncharged Lys residues were placed tightly packed in the simulation box of 9.62 × 9.37 × 10.42 nm^3^. This system was then equilibrated with a 100 ns production run, and the final configuration was used as the initial system for the first simulation at acidic pH = 3.

Eight simulations were performed in series of 100 ns each [pH3 → pH11→ pH3 …] for a total of 0.8 µs. L2 molecules at pH = 3 were obtained by adding a third H atom to the NH_2_ group of the Lys sidechain (NH_2_ → NH_3_^+^), and vice versa: the molecules at pH = 11 were obtained from those at pH = 3 by eliminating a H atom (NH_3_^+^→NH_2_).

The initial configuration of the system (64 molecules of L2) at pH = 11 was obtained from the last one (after 100 ns) of the previous simulation at pH = 3. In the simulation box, in addition to the solvation water molecules, an equal number of Na^+^ and Cl^−^ ions were added randomly to reproduce the experimental ionic strength. The initial monolayer configurations at pH = 3 were obtained from the previous configuration at pH = 11 after 100 ns of simulation. In the simulation box, in addition to the solvation water molecules, 512 Cl^−^ ions were added randomly to neutralize the positive charges of the 512 Lys present. The average number of water solvation molecules for the various simulations was approximately 30,000.

The S−Au bond was mimed by constraining the Z-coordinates of the sulfur atoms of lipoic moiety at 0.268 nm far from the Au layer by means of a harmonic potential with force constants equal to 1000 kJ mol^−1^ nm^−2^. The positions of each Au atom were harmonically constrained to their starting values with a force constant equal to 10,000 kJ mol^−1^ nm^−2^.

All of the parameters and simulation data were analyzed in the last 10 ns of each simulation when the systems were equilibrated.

The densities of peptide, water, and ions were calculated using the Gromacs *density* tool. The solvent accessible surface area (SASA) was obtained using the Gromacs *sasa* tool. The secondary structure was analyzed using the Gromacs *dssp* tool [39]. The mass variation after each cycle was calculated by comparing the weight on the surface at pH = 3 and at pH = 11 due to the peptides, to the ions eventually present in the monolayer, and to the water molecules near the peptides (within 5 Å from the peptides).

A visual molecular dynamics (VMD) program [40] was used for the visualization of structures.

## 4. Conclusions

The stimuli-response behavior of an assembly of a twenty-one-residue-long GA IV analog (L2) has been investigated in detail by means of both experimental analysis and molecular dynamic simulations.

Experimentally, it was observed that changes from pH = 3 to 11 in the systems were observed to lead to a strong variation in the thickness of the layer and in a variation in the mass of the system, with a mass depletion occurring when the pH drops from an acidic to a basic pH. This is in line with the results already found with a system constituted by a peptide that is half the length of L2, thus having the same features but being constituted by only 11 amino acids. Surprisingly, with L2, a hysteresis over pH cycling is observed, leading to a decrease in adsorbed water mass. MD simulations performed on a system consisting of a peptide monolayer chemisorbed on a gold surface and with a cyclically altered pH from pH = 3 to pH= 11, and vice versa, have been able to explain four times, at the molecular level, this interesting experimental result.

Simulations demonstrated that the observed changes in the monolayer are due to the rearrangements of Lys side chains that are pH sensitive, involving very little variation in the secondary structure of the peptides. At acid pH, the Lys side chains, indeed, are charged, so that at low pH values, peptides are surrounded by water molecules and counterions; in contrast, at basic pH, Lys amine groups are neutral, and, therefore, hydrophobicity becomes prevalent; consequently, peptides tend to aggregate, depleting water from the surrounding areas, and therefore loosing mass. The slight variation in the secondary structure involves a decrease in the helical content and an increase in β-sheets content when performing subsequent pH cycling. These local variations, in turn, lead to a change in the assembly features, with a formation of even smaller aggregates. Furthermore, this hysteresis process is mainly due to the first half of L2, that is, the peptide portion closer to the surface, while the second half, further from the surface, remains almost unaffected by the ongoing pH cycles.

The results indicate that L2 on gold behaves like a “two floor” peptide, in which each of the halves assumes a different rearrangement.

This study represents a proof of concept on a detailed analysis of peptide assembly on a surface to evaluate, at molecular level, its robustness under use. Even if this system could be employed to develop, for example, electronic devices, the great pH interval in which it works renders it not immediately applicable for biomedical applications. However, the protocol could be useful for evaluating systems with other more tuned substitutions, such as His instead of Lys, and thus responsiveness in a shorter pH range, which is more compatible for drug delivery or biomedical applications. 

## Figures and Tables

**Table 1:** QCM-D and SPR data for the peptide L2 adsorbed on gold-coated sensor surfaces. Data were taken after 18 h of incubation in ethanol.

	∆F (Hz)	∆D (10^−6^)	Weight (ng/cm^2^)	Layer Thickness from SPR (nm)
L2	−60.2 ± 9.0	1.5 ± 0.5	1065.4 ± 159.7	5.66 ± 0.28

## Data Availability

Data will be made available on request.
